# Poly-Pharmacologic Disruption of the Proliferative-to-Mesenchymal Fate Branch Point Reverses EndMT and Pulmonary Hypertension

**DOI:** 10.21203/rs.3.rs-9306408/v2

**Published:** 2026-07-23

**Authors:** Dinesh Bharti, Tetiana Kolodiazhna, Sedat Kacar, Mathews V Varghese, Takanori Sano, Sruthi Radhakrishnan, Joel James, AJ Hinkle, Maki Niihori, Olga Rafikova, Alexander V. Statsyuk, Ruslan Rafikov

**Affiliations:** 1. Department of Medicine, Division of Pulmonary, Critical Care, Sleep & Occupational Medicine, Indiana University, Indianapolis, Indiana 46202; 2. Department of Pharmacological and Pharmaceutical Sciences, College of Pharmacy, University of Houston, Houston, TX, 77204-5039

**Keywords:** endothelial-to-mesenchymal transition, fate branch point, polypharmacology, kinome profiling, vascular remodeling, pulmonary arterial hypertension, single-cell RNA sequencing

## Abstract

Endothelial-to-mesenchymal transition (EndMT) drives the vascular remodeling in pulmonary arterial hypertension (PAH), yet the regulators that commit endothelial cells to this fate and whether they are pharmacologically addressable remain poorly defined. We report TK22, a small molecule that selectively erases the EndMT-competent endothelial state and reverses experimental pulmonary hypertension. TK22 was developed by structure-based design as an ATP-competitive CDK5 inhibitor (IC_50_ 181 nM). Unbiased profiling across 394 kinases, however, revealed that TK22 inhibits the interphase cell-cycle kinases CDK2/3/4/6 node as well as a pro-mesenchymal node of AMPK-related kinases (NUAK1 and SIK1/2/3) together with PDGFRβ and MLK3, placing its activity at the intersection of proliferative and EndMT control rather than at a single node. Owing to this dual-node action, TK22 produced a strikingly clean cellular phenotype. In primary murine lung endothelial cells, TK22 reversed TGF-β1-induced EndMT across morphological, proteomic, and functional readouts, normalizing α-SMA, SM22, and Calponin, restoring angiogenic tube formation, abolishing acquired smooth-muscle-like contractility, and returning TGF-β1-driven hyperproliferation to baseline without suppressing normal endothelial growth. Single-cell RNA sequencing resolved the basis of this precision: TGF-β1 redirected a cycling Mki67^+^ endothelial subpopulation into an EndMT-committed state (Tnnt2, Ptgs2, Serpine1) and induced an associated metabolic-stress program (Egln3, Pfkfb3, Pdk1, Bnip3). Cross-validated PHATE and Monocle3 trajectory inference defined a directional proliferative-to-mesenchymal fate transition, and kernel density estimation revealed a stable EndMT cell-state attractor. TK22 selectively eliminated the EndMT-competent and metabolic-stress subpopulations while leaving the remaining endothelial landscape intact. This population-level precision of TK22 is consistent with its dual engagement of the anti-cell-cycle and anti-mesenchymal kinase nodes. In the Su5416/hypoxia rat PH model, TK22 normalized right ventricular systolic pressure, Fulton index, and cardiac function. Together, these findings reframe pharmacologic EndMT control: inhibition at the proliferative-to-mesenchymal fate branch point, through coordinated suppression of cell-cycle and mesenchymal kinases, achieves high selectivity for erasing a pathological endothelial fate state. These findings propose TK22 as a mechanistically precise agent for EndMT-driven vascular disease and establish single-cell trajectory analysis as an essential readout of cellular target engagement and cell-state precision.

## Introduction

Pulmonary arterial hypertension (PAH) is a progressive vascular disease in which heterocellular remodeling of the pulmonary arteries drives a relentless rise in vascular resistance, right ventricular failure, and death^1,2^. A central and increasingly appreciated contributor to this remodeling is endothelial-to-mesenchymal transition (EndMT), a process in which endothelial cells relinquish their identity and acquire mesenchymal, migratory, and contractile properties that fuel fibroproliferative narrowing of the vessel wall^3,4^. EndMT is not a graded loss of endothelial character but a discrete fate decision: cells commit to a mesenchymal program, remodel their cytoskeleton and adhesive contacts, and stabilize in a new transcriptional state^5^. TGF-β1, the most extensively studied isoform, activates canonical Smad2/3/4 signaling and non-canonical PI3K/AKT and NF-κB cascades that converge on the transcriptional up-regulation of mesenchymal master regulators such as Twist, Snail, Slug, and ZEB^6^. Elevated circulating TGF-β1 in idiopathic and heritable PAH correlates with adverse outcomes, underscoring the pathological weight of this axis in disease progression^7^. Yet the identity of the regulators that commit an endothelial cell to the mesenchymal fate downstream of TGF-β1, and whether that commitment step is pharmacologically correctable, has remained poorly defined.

A fundamental obstacle is that fate commitment is unlikely to be governed by any single kinase. The proliferative-to-mesenchymal transition sits at the intersection of two cellular programs: the cell-cycle machinery that maintains the cycling endothelial pool from which EndMT emerges, and the mesenchymal-signaling machinery that executes cytoskeletal and adhesive reprogramming. This architecture predicts that durable, cell-type-selective control of EndMT would be achieved not by silencing one node but by coordinated inhibition at the branch point where these programs meet, a polypharmacology^8^ that is difficult to reach with single-target design and easy to misattribute to whichever target motivated the chemistry.

TK22 emerged from precisely such a single-target starting point. It was developed by structure-based drug design as an ATP-competitive inhibitor of cyclin-dependent kinase 5 (CDK5), an atypical CDK activated by the non-cyclin cofactors p35 and p25 that has been implicated in cytoskeletal remodeling, fibroblast activation, cancer metastasis, and cardiovascular remodeling^9–15^, and it inhibits recombinant CDK5 with an IC_50_ of 181 nM. However, when TK22 was profiled unbiasedly across 394 kinases, CDK5 was not among its principal targets. Instead, TK22 potently and consistently engaged two functional nodes: the interphase cell-cycle kinases CDK2, CDK3, CDK4, and CDK6, and a pro-mesenchymal node dominated by the AMPK-related kinases NUAK1 and SIK1/2/3 together with the receptor and stress kinases PDGFRβ and MLK3. This profile places TK22’s activity at the intersection of proliferative and mesenchymal control, at the proliferative-to-mesenchymal fate branch point itself, rather than at any single kinase, and reframes CDK5 as the design anchor that, in broad profiling, proved to influence a wider, mechanistically coherent inhibitory program. Here we use TK22 as a pharmacologic probe to test whether disrupting this dual-node branch point reverses EndMT and its vascular consequences.

## Results

### EndMT markers are upregulated in the experimental PH model

**Figure 1.**
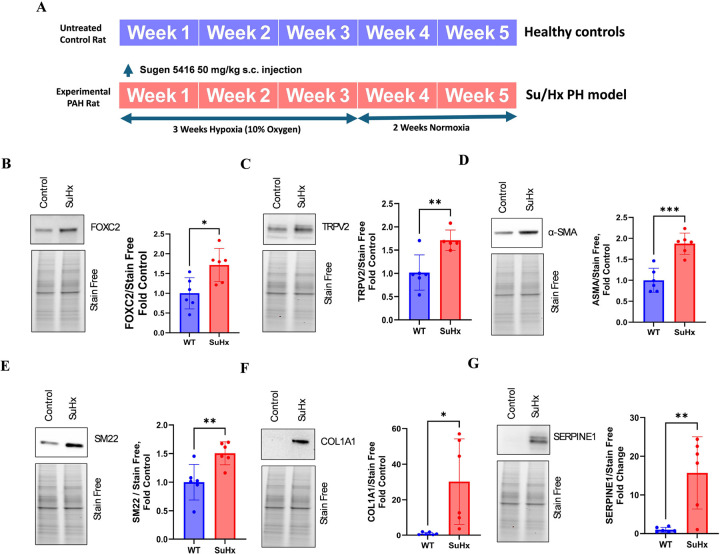
EndMT-associated markers are elevated in the SuHx rat model of PAH. Lung lysates from control and Su5416/hypoxia (SuHx)-treated rats were subjected to SDS-PAGE and immunoblotting with Stain-Free total-protein normalization. (A) Schematic of the experimental setup for protein lysates from control and experimental PAH rats. (B) FOXC2, a transcription factor associated with EndMT and epithelial-mesenchymal plasticity, was upregulated in the SuHx group. (C) TRPV2, a mechanosensitive Ca^2^^+^ channel implicated in fibrotic remodeling, was significantly increased in SuHx lungs. (D,E) α-smooth muscle actin (α-SMA) and SM22, canonical mesenchymal markers, were markedly elevated in SuHx lungs, consistent with active vascular remodeling. (F,G) COL1A1, a non-myogenic fibroblast marker of EndMT, and SERPINE1, a biomarker of tissue fibrosis and vascular remodeling, were also significantly upregulated in SuHx lungs relative to control. Representative immunoblots are shown alongside quantification. Data are mean ± SD (n = 6 per group); unpaired two-tailed Student’s t-test. *p < 0.05; **p < 0.01; ***p < 0.001.

To evaluate the role of EndMT in vascular remodeling in PAH, untreated control and Su5416/Hypoxia-treated animal lungs were excised and processed for western blotting using EndMT-specific markers. These lung lysates were taken from pre-verified experimental PAH models^16^ (schematic in Fig. 1A). As expected, Su/Hx lung samples exhibited significant upregulation of FOXC2 (an EndMT/EMT-associated transcription factor), TRPV2 (a mechanosensitive Ca^2^^+^ channel implicated in fibrotic and remodeling responses), α-smooth muscle actin (α-SMA) and Transgelin (TAGLN/SM22) (myogenic markers associated with EndMT), collagen type I alpha 1 chain (COL1A1; a non-myogenic fibroblast marker of EndMT), and Serpin family E member 1 (SERPINE1/PAI-1; an activator and promoter of EndMT), together indicating that EndMT is an active component of vascular remodeling and PAH disease progression (Fig. 1B–G).

### Structure-based design of TK22 and unbiased kinome profiling reveal a dual-node inhibitory program

**Figure 2.**
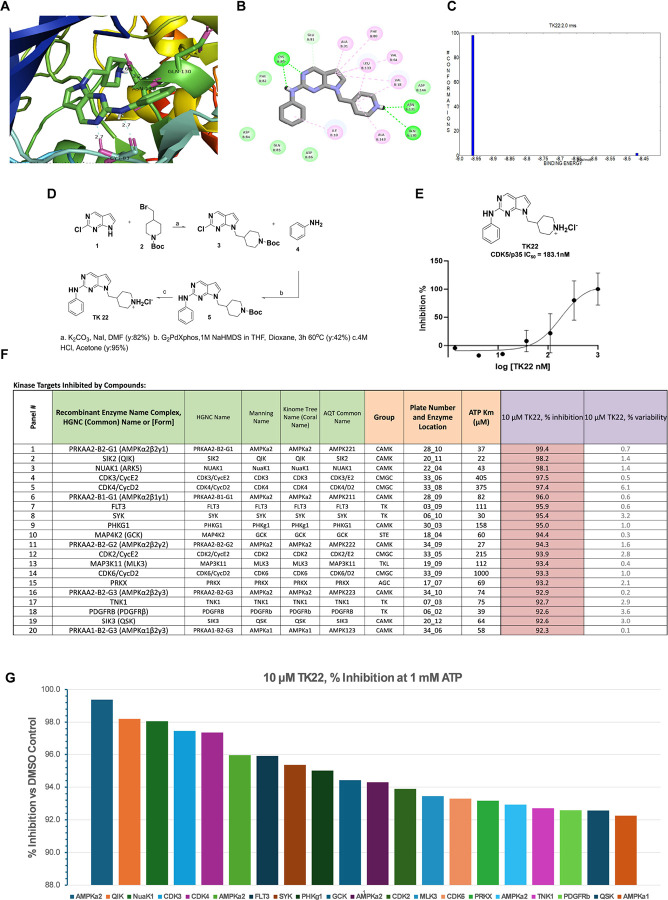
Structure-based design, synthesis, and biochemical evaluation of TK22 and unbiased kinome profiling reveal a dual-node inhibitory program. (A) Three-dimensional binding pose of TK22 docked in the ATP-binding cleft of CDK5/p25 (PDB: 7VDP), visualized in PyMOL, illustrating hydrogen-bond interactions (dashed lines, distances in Å) with hinge-region residues Cys83, Gln130, and Asn131. (B) Two-dimensional interaction map (Discovery Studio Visualizer) depicting conventional hydrogen bonds (green) and hydrophobic/π-alkyl contacts (pink) with Phe80, Ala31, Val18, Val64, Leu133, Ile10, and Ala143, consistent with ATP-competitive engagement of the design target. (C) Binding-energy histogram of 100 docking conformations (Lamarckian Genetic Algorithm, AutoDock 4); the predominant cluster at −8.79 kcal/mol confirms a single, well-defined binding mode. (D) Three-step synthetic route to TK22. Step a: N-alkylation of 2-chloro-7H-pyrrolo[2,3-d]pyrimidine (1) with tert-butyl 4-(bromomethyl)piperidine-1-carboxylate (2). Step b: palladium-catalyzed Buchwald-Hartwig amination with aniline (G2PdXPhos, dioxane). Step c: acid-mediated Boc deprotection (4 M HCl) furnishing TK22 as the hydrochloride salt. Structure and purity (≥97% by HPLC) were confirmed by ^1^H/^1^^3^C NMR and high-resolution mass spectrometry. (E) Dose-response inhibition of recombinant human CDK5/p35 by TK22 (Kinase-Glo Plus, 100 μM ATP, Histone H1; 7-point series 1 μM-1.37 nM), fitted by four-parameter logistic regression, yielding an IC_50_ of 181.3 nM (mean ± SD of technical replicates). (F,G) Unbiased kinome profiling of TK22 across 394 recombinant kinases (10 μM TK22, 1 mM ATP; % inhibition vs. DMSO). (F) Ranked percentage inhibition for the top kinases, colored by Manning family; CDK5 complexes (ranked 96th and 113th, 42–54% inhibition) are highlighted to show that the design anchor is not a principal target. (G) The strong hits converge on two functional nodes: an interphase cell-cycle node (CDK2, CDK3, CDK4, CDK6; 93–97% inhibition) and a pro-mesenchymal AMPK-related kinase node (NUAK1, SIK1/2/3) with PDGFRβ and MLK3 (90–98% inhibition), placing TK22’s activity at the proliferative-to-mesenchymal fate branch point.

TK22 was designed as an isosteric analogue of previously reported selective CDK5 inhibitors, with the objective to simplify the synthesis, such that large quantities of TK22 can be easily produced for in vivo studies. TK22 achieves an ATP-competitive binding mode in CDK5, by (1) incorporating hydrogen-bonding features capable of anchoring the compound within the hinge/polar region of the ATP cleft and (2) presenting hydrophobic functionality to complement the lipophilic subpocket adjacent to the catalytic site. Docking into the CDK5/p25 ATP-binding pocket predicted that TK22 adopts a canonical ATP-site pose well accommodated within the active-site cleft (Fig. 2A–B). In the top-ranked binding mode, TK22 forms three conventional hydrogen bonds with Cys83, Gln130, and Asn131 (chain B), supporting stable polar anchoring, and is further stabilized by extensive hydrophobic contacts with Phe80, Ala31, Val64, Val18, Ile10, Leu133, and Ala143. Additional close contacts with Glu81, Asp84-Asp86, Gln85, Phe82, and Asp144 indicate that TK22 is embedded within a mixed polar-hydrophobic microenvironment. Collectively, the predicted pose and interaction network support an ATP-competitive binding mode for TK22 in CDK5, providing the structural rationale for its design.

**TK22** was synthesized via a modular three-step route (Fig. 2D) starting from 2-chloro-7H-pyrrolo[2,3-d]pyrimidine (**1**), which was N-alkylated with tert-butyl 4-(bromomethyl)piperidine-1-carboxylate (**2**) to yield intermediate **3**^17^. Compound **3** was then subjected to a palladium-catalyzed Buchwald–Hartwig amination with aniline, using G2PdXPhos as catalyst, to yield compound **5**^18^. The Boc-protected analog **5** was deprotected using 4 M HCl in dioxane^19^ to afford TK22 as an amine hydrochloride salt, in an overall yield of 47% (Supplemental Fig. 1–5). The inhibitory potency of TK22 against its design target was evaluated using the Kinase-Glo Plus luminescent assay with recombinant human CDK5/p35 complex. TK22 was tested in a 7-point dose-response format from 1 μM to 1.37 nM (Fig. 2E) and inhibited CDK5 with an IC_50_ of 181.3 nM.

To define the full range of target kinases for TK22, we profiled TK22 in an unbiased manner across 394 kinases at 10 μM concentration in the presence of 1 mM ATP, thereby conducting the assay at physiological ATP concentrations. (Fig. 2F–G) (Supplemental data 2). DMSO was used as a negative control. This broad profiling revealed that TK22 is not a selective CDK5 inhibitor: in fact, CDK5 was a comparatively weak target, with the CDK5/p35 and CDK5/p25 complexes inhibited by only 54% and 42%. Instead, TK22 inhibited 72 kinases by more than 70% of DMSO control activity, of which 27 exceeded 90% inhibition (Supplemental data 2). Critically, these latter strong hits were not randomly scattered but converged on two mechanistically coherent functional nodes (Fig. 2F–G). The first is an interphase cell-cycle node: TK22 potently inhibited CDK3 (97%), CDK4 (97%), CDK2 (94%), and CDK6 (93%), the kinases^20–22^ that drive the cycling endothelial population from which EndMT emerges, placing all four among the strongest hits in the panel. The second is a pro-mesenchymal node built around the AMPK-related kinase (ARK) family: NUAK1 (98%) and the salt-inducible kinases SIK2 (98%), SIK3 (93%), and SIK1 (90%) were among the very top hits, together with the receptor tyrosine kinase PDGFRβ (93%) and the MAP3K, MLK3 (MAP3K11; 93%). These kinases map onto TGF-β transcriptional output, YAP/SMAD fibrogenic reinforcement, non-SMAD MAPK/JNK signaling, and PDGF/TGF-β vascular-remodeling crosstalk, and the AMPK-related component connects directly to the metabolic-stress program that accompanies EndMT. TK22 thus acts as a dual-node polypharmacology agent whose activity sits at the intersection of proliferative and mesenchymal control, the proliferative-to-mesenchymal fate branch point, rather than at CDK5 or any single kinase. This profile reframes CDK5 as the design anchor that, in broad profiling, proved to influence a broader, more coherent inhibitory program, and nominates TK22 as a drug lead or chemical probe for pharmacological characterization in PAH-relevant models.

### TGF-β1 drives progressive, stage-dependent EndMT in primary mLECs, which is reversed by TK22

**Figure 3.**
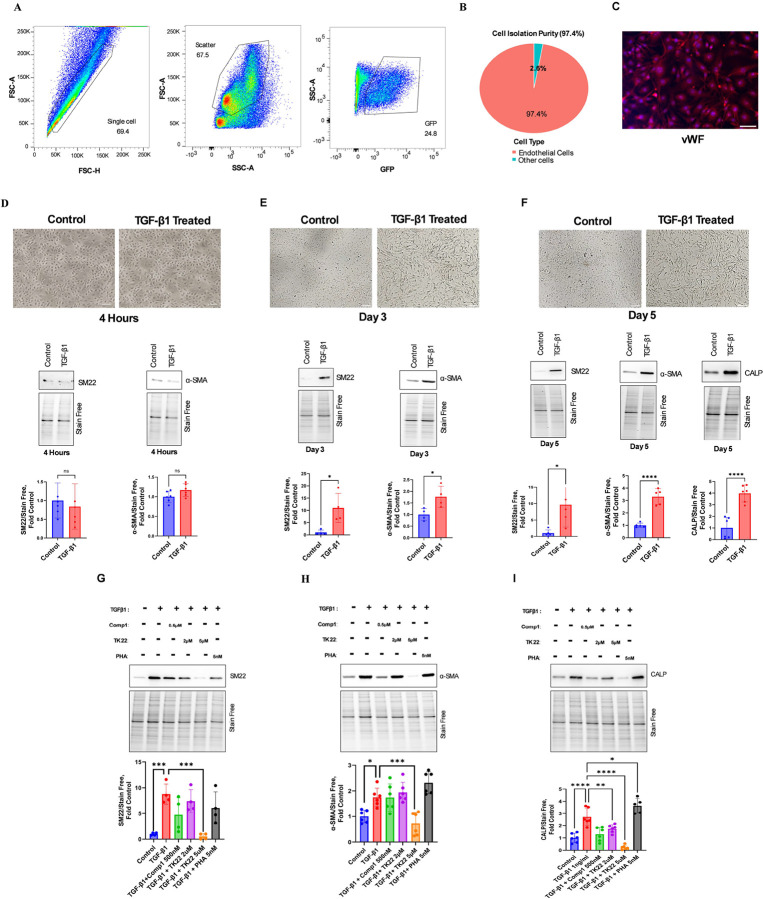
Isolation of GFP-expressing murine lung endothelial cells, stage-dependent EndMT induction, and its reversal by TK22. Primary mLECs were isolated from Tie2-GFP transgenic mice by FACS, with wild-type C57BL/6 lung cells as GFP-negative gating controls. (A) Sequential gating: FSC-A vs. FSC-H (singlets, 69.4%), FSC-A vs. SSC-A (viable, 67.5%), SSC-A vs. GFP (GFP^+^ endothelial cells, 24.8%). (B) Post-sort purity of 97.4% endothelial enrichment (2.6% non-endothelial). (C) Endothelial identity confirmed by von Willebrand factor (vWF; red) immunofluorescence with DAPI (blue). Scale bar, 50 μm. (D-F) GFP-mLECs treated with TGF-β1 (1 ng/ml) for 4 hours, 3 days, or 5 days, with phase-contrast morphology and Stain-Free-normalized immunoblots for early (SM22, α-SMA) and mature contractile (Calponin, CALP) markers. (D) At 4 hours, cells retained cobblestone morphology and baseline SM22/α-SMA. (E) By day 3, cells adopted an elongated morphology with significant SM22 and α-SMA upregulation. (F) By day 5, a fully transitioned spindle-shaped phenotype with robust SM22, α-SMA, and CALP. The 5-day induction was used for subsequent experiments. (G-I) GFP-mLECs treated with TGF-β1 (1 ng/ml, 5 days) alone or with the indicated kinase inhibitors: compound 1 (Comp1; 500 nM, promiscuous covalent inhibitor), TK22 (2 μM or 5 μM), or PHA-793887 (PHA; 5 nM, commercial CDK5 inhibitor). Representative immunoblots and quantification of SM22 (G), α-SMA (H), and CALP (I) normalized to Stain-Free total protein. TK22 at 5 μM produced the most pronounced reduction of SM22 and CALP and was the only inhibitor to significantly reduce α-SMA; PHA-793887 unexpectedly elevated CALP, dissociating EndMT reversal from single-target CDK5 inhibition. Data are mean ± SD (n = 5–6 per group); For grouped comparisons unpaired two-tailed Student’s t-test, for multiple comparisons one-way ANOVA with Bonferroni correction. ns, not significant; *p < 0.05; **p < 0.01, ***p < 0.001, ****p < 0.0001.

Single-cell lung suspensions from Tie2-GFP mice (Tg(TIE2GFP)287Sato/J) were subjected to FACS to isolate GFP-positive murine lung endothelial cells (mLECs), with WT C57BL/6 samples used as GFP-negative gating controls (Fig. 3A). Post-sort analysis demonstrated a highly enriched endothelial population with 97.4% purity (Fig. 3B), and endothelial identity was confirmed by robust immunocytochemical staining for vWF (Fig. 3C). To determine optimal EndMT-induction conditions, GFP-mLECs at passage 4 were exposed to 1 ng/ml TGF-β1 for 4 hours, 3 days, and 5 days, and assessed for early (α-SMA, SM22) and mature (Calponin) EndMT markers^23^ (Fig. 3D–F). At 4 hours, cells showed no discernible morphological change or difference in α-SMA and SM22 relative to controls (Fig. 3D). By Day 3, cells adopted an elongated morphology with significantly elevated α-SMA and SM22 (Fig. 3E). Extended exposure (Day 5) produced a fully transitioned, spindle-shaped, fibroblast-like phenotype with robust accumulation of α-SMA, SM22, and the mature contractile marker Calponin (Fig. 3F).

To test whether the TK22 branch-point program reverses this transition, GFP-mLECs were induced with 1 ng/ml TGF-β1 for 5 days in the presence or absence of TK22 (2 μM and 5 μM), benchmarked against PHA-793887 (a commercial CDK5 inhibitor) and Compound-1^24^ (a promiscuous kinase inhibitor). Notably, the comparators dissociate cell-fate reversal from single-target CDK5 inhibition. All three compounds attenuated the TGF-β1 mesenchymal phenotype morphologically, but their effects on EndMT markers diverged. SM22, upregulated ~12-fold by TGF-β1, was most strongly reduced by TK22 at 5 μM (Fig. 3G). α-SMA was significantly diminished only in the TK22 (5 μM) group, with no notable change under PHA-793887 or Compound-1 (Fig. 3H). The mature contractile marker Calponin (CALP) was reduced by TK22 (2 μM and 5 μM) and Compound-1, with maximal inhibition at 5 μM TK22, whereas PHA-793887 unexpectedly elevated CALP (Fig. 3I). That the dedicated CDK5 inhibitor PHA-793887 failed to reproduce, and in the case of CALP opposed, TK22’s marker reversal argues that TK22’s efficacy does not derive from CDK5 inhibition but from its broader dual-node engagement. TK22 at 5 μM was carried forward as the most effective condition.

### TK22 reverses EndMT, normalizes endothelial proliferation, and restores endothelial function

**Figure 4.**
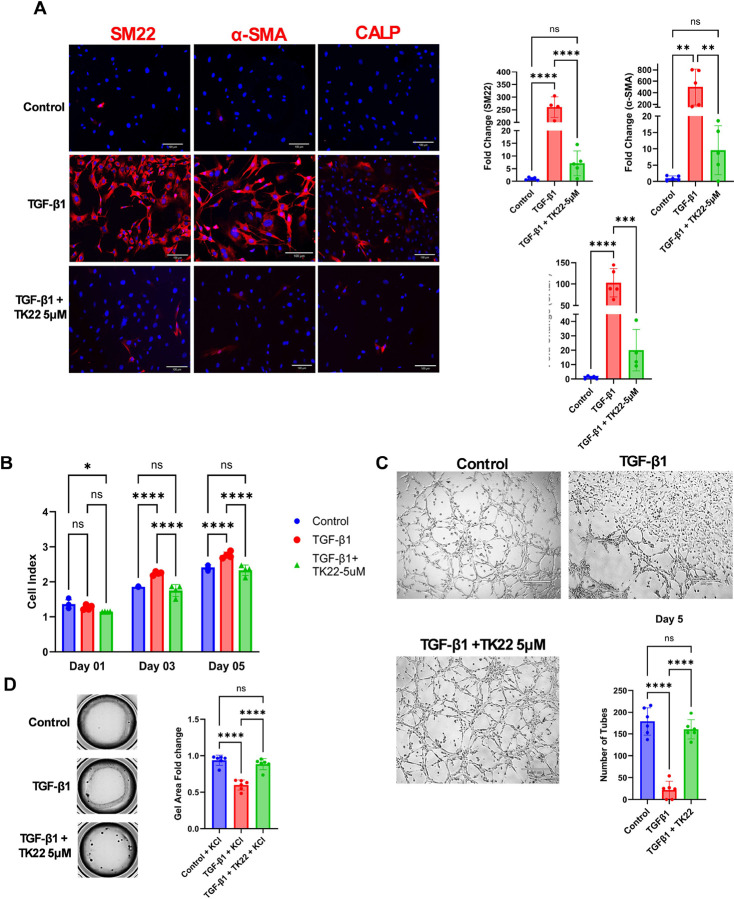
TK22 reverses TGF-β1-induced EndMT, normalizes proliferation without suppressing baseline growth, and restores endothelial function. GFP-mLECs were treated with TGF-β1 (1 ng/ml, 5 days) alone or with TK22 (5 μM). (A) Immunofluorescence for SM22, α-SMA, and CALP (red) with DAPI (blue). TGF-β1 increased all three markers, each significantly reduced by TK22. Scale bar, 100 μm. (B) Proliferation (CCK-8 cell index) at days 1, 3, and 5. TGF-β1 significantly increased proliferation by days 3 and 5; TK22 suppressed this increase, restoring proliferation to control baseline while sparing normal endothelial growth. (C) Matrigel tube-formation assay at day 5 (representative micrographs, scale bar 250 μm, and tube-number quantification). TGF-β1 severely impaired tube formation; TK22 restored angiogenic capacity toward control. (D) Collagen gel contraction with 80 mM KCl over 24 hours. TGF-β1-treated cells showed significant gel compaction reflecting acquired smooth-muscle-like contractility; TK22 abolished this contraction. Data are mean ± SD (n = 6 per group). Proliferation analyzed by two-way ANOVA with Tukey’s multiple-comparisons test; angiogenesis and gel contraction by one-way ANOVA with Bonferroni correction. ns, not significant; *p < 0.05; **p < 0.01; ***p < 0.001; ****p < 0.0001.

The EndMT-reversing activity of TK22 was confirmed by immunocytochemistry: TGF-β1 increased the number of cells positive for SM22, α-SMA, and CALP, indicating active EndMT, while TK22 at 5 μM significantly reduced marker-positive cells (Fig. 4A). Proliferation of untreated control, TGF-β1, and TGF-β1 + 5 μM TK22 groups was assessed at days 1, 3, and 5 (Fig. 4B). No differences were seen at Day 1. By Days 3 and 5, TGF-β1 significantly increased proliferation relative to controls, consistent with EndMT-associated cellular activation; TK22 suppressed this increase, restoring proliferation to control baseline. Importantly, TK22 returned TGF-β1-driven hyperproliferation to baseline without suppressing the normal endothelial growth of untreated cells, the behavior expected of coordinated cell-cycle-node inhibition acting on the pathologically activated pool rather than of a general cytostatic agent.

Following identification of TK22 as the most effective condition, its impact on endothelial function was evaluated. On Matrigel, untreated control endothelial cells displayed extensive tube-like structures, whereas TGF-β1-treated cells showed markedly reduced tube formation, indicating loss of endothelial function with phenotypic transition; TK22 restored angiogenic capacity toward control levels (Fig. 4C). Cells were also assessed for collagen-gel contraction in response to KCl stimulation (Fig. 4D). At 0 and 3 hours, no group showed significant contraction with or without 80 mM KCl. At 24 hours, TGF-β1-induced cells showed a significant decrease in gel area, demonstrating acquired smoothmuscle-like contractility characteristic of EndMT; this contraction was absent in untreated control and TK22-treated groups, indicating that TK22 abolished the acquisition of contractile mesenchymal behavior (Fig. 4D). Together with the western blotting and immunocytochemistry data, these functional readouts confirm that TK22 reverses TGF-β1-induced EndMT across morphological, proteomic, and functional axes while preserving normal endothelial identity and growth.

### Single-cell RNA sequencing resolves an EndMT fate transition and its selective reversal by TK22

**Figure 5.**
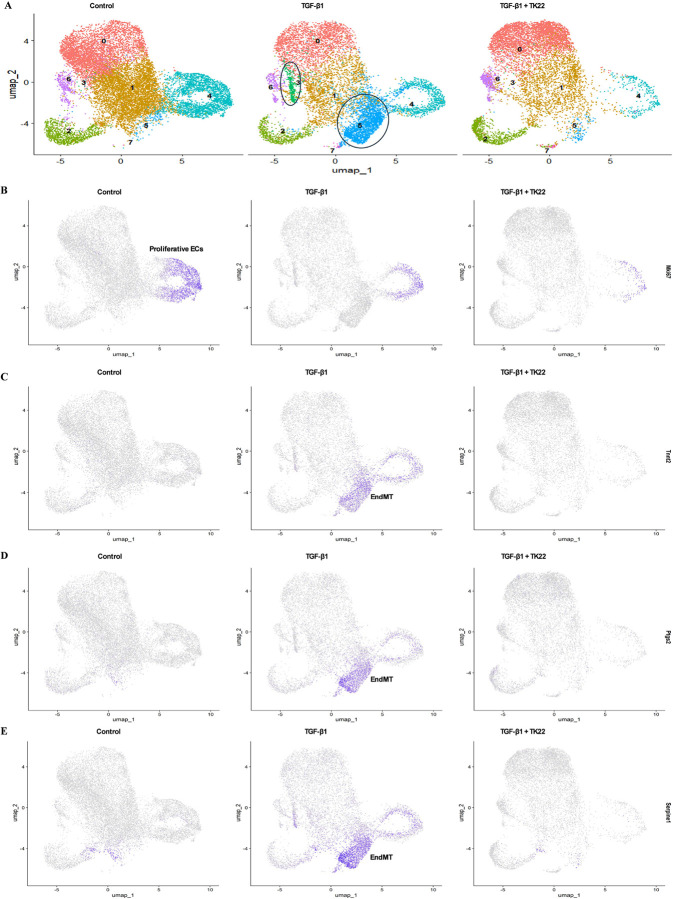
Single-cell RNA sequencing identifies a proliferative-to-EndMT fate transition that is selectively reversed by TK22. GFP-mLECs were profiled by scRNA-seq (10x Genomics Flex) under untreated control, TGF-β1, and TGF-β1 + TK22 conditions. After QC and anchor-based integration, clusters were resolved by unsupervised Seurat clustering and visualized by UMAP. (A) UMAP per condition, colored by cluster. TGF-β1 enriched clusters 5 and 3 (circled); TK22 reduced these TGF-β1-expanded clusters, restoring a control-like distribution. (B) Feature plots of Mki67; Cluster 4 is the principal cycling subpopulation. Under TGF-β1, Mki67^+^ representation in Cluster 4 declines as cells accelerate into the EndMT state (Cluster 5) rather than arresting; TK22 selectively reduces Cluster 5. (C-E) Feature plots of the EndMT-associated transcripts Tnnt2 (C), Ptgs2 (D), and Serpine1 (E), marking cytoskeletal remodeling, inflammatory activation, and fibrinolytic suppression. These were enriched in Cluster 5 and extended into the Cluster 4 region under TGF-β1, indicating that proliferative ECs initiate EndMT reprogramming as they transition; TK22 abolished expression of all three across the UMAP.

To resolve how the TK22 branch-point program reshapes endothelial cell-state dynamics during TGF-β1-driven EndMT, we performed scRNA-seq on GFP-sorted primary mLECs cultured under untreated conditions, TGF-β1 stimulation, or TGF-β1 plus TK22 (5 μM). After quality control and dimensionality reduction, endothelial cells segregated into transcriptionally distinct clusters spanning proliferative, quiescent, inflammatory, transitional, and mesenchymal-like programs (Fig. 5A). Two clusters were selectively modulated by TGF-β1 and restored toward control representation by TK22: a highly proliferative subpopulation (Cluster 4) and an EndMT-associated subpopulation (Cluster 5).

Cluster 4 showed strong expression of the cell-cycle gene Mki67, marking it as the principal cycling endothelial subpopulation (Fig. 5B). Cluster 5 was selectively expanded under TGF-β1 and characterized by elevated Tnnt2, Ptgs2, and Serpine1 (Fig. 5C–E), transcripts linked to cytoskeletal contractile remodeling, inflammatory activation, and suppression of fibrinolysis, and recently identified as EndMT-associated markers by multi-omics analysis^25^. Critically, these EndMT-linked transcripts were not confined to Cluster 5 but extended into the Cluster 4 region under TGF-β1 (Fig. 5C–E), indicating that cycling endothelial cells initiate mesenchymal transcriptional reprogramming before fully exiting the proliferative state. The net reduction in steady-state Cluster 4 representation under TGF-β1 therefore reflects not suppressed proliferation but accelerated progression of cycling ECs into an EndMT fate (Cluster 5), a routing that directly implicates the two TK22 nodes, cell-cycle and pro-mesenchymal kinases, at the point where a proliferative cell commits to a mesenchymal fate.

**Figure 6.**
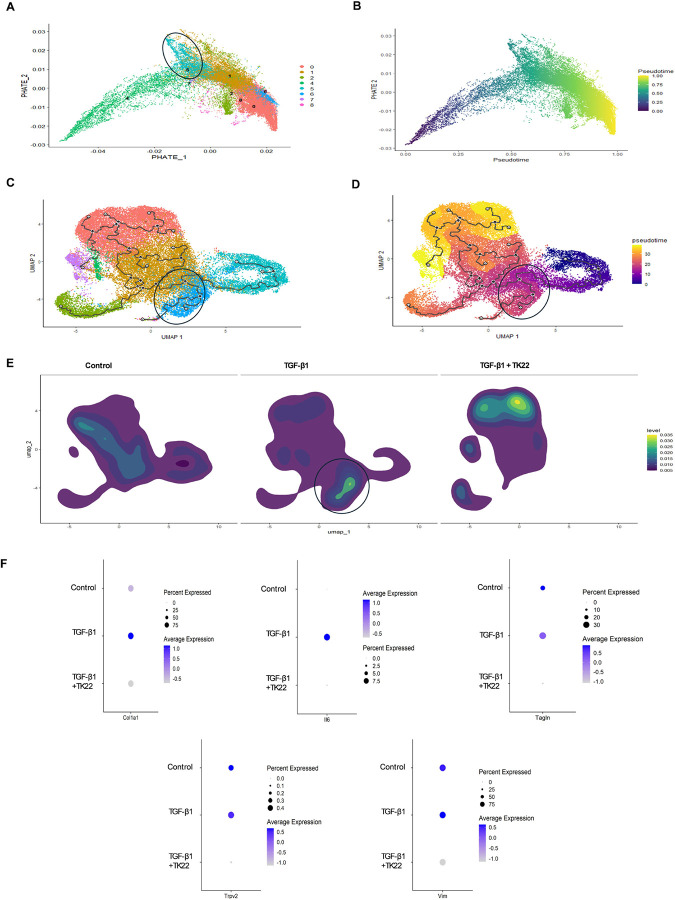
Trajectory inference and cell-state density analysis define a directional proliferative-to-EndMT fate transition and a stable EndMT attractor abolished by TK22. (A,B) PHATE dimensionality reduction of the integrated dataset. (A) PHATE colored by Seurat cluster, revealing a continuous manifold; proliferative Cluster 4 anchors the early trajectory with EndMT Cluster 5 (circled) immediately downstream. (B) PHATE colored by pseudotime, showing directional progression from proliferative states at low pseudotime toward transitional states at high pseudotime, consistent with Cluster 4 as the trajectory origin. (C,D) Monocle3 trajectory overlaid on UMAP. (C) Cells colored by cluster with the principal graph and nodes; the trajectory originates in proliferative Cluster 4 and diverges early toward either the main endothelial identity or the EndMT Cluster 5 (circled). (D) The same UMAP colored by Monocle3 pseudotime, with Cluster 5 (circled) at high pseudotime, corroborating a proliferative-to-EndMT sequence under TGF-β1. (E) Kernel density estimation of cell distributions across UMAP for each condition. TGF-β1 produces a high-density EndMT attractor (circled) absent in controls and markedly diminished by TK22, reflecting selective elimination of the EndMT-competent subpopulation. (F) Dot plots of average scaled expression (color) and percentage of expressing cells (dot size) for EndMT-associated genes Col1a1, Il6, Tagln, Trpv2, and Vim across conditions; each is elevated under TGF-β1 and reduced by TK22, reinforcing coordinated transcriptomic reversal of EndMT.

To characterize the directional dynamics of this transition, we applied two complementary trajectory-inference approaches. PHATE was used to visualize the global topology of the transcriptional manifold without a user-defined starting node (Fig. 6A–B). The PHATE embedding revealed a continuous, branching manifold in which Cluster 4 anchored the origin of the principal trajectory arm and the EndMT Cluster 5 occupied an immediately adjacent position (Fig. 6A). Coloring by pseudotime (Fig. 6B) placed proliferative Cluster 4 cells at the lowest pseudotime and EndMT-arm cells at progressively higher pseudotime, consistent with a directional proliferative-to-mesenchymal transition. The PHATE-inferred root was then used to initialize Monocle3 pseudotime for a biologically grounded, internally consistent starting point.

Monocle3 corroborated the PHATE topology (Fig. 6C–D). The principal graph overlaid on the UMAP (Fig. 6C) showed the inferred trajectory originating within proliferative Cluster 4 and diverging early into two branches: one extending toward the broader endothelial-identity landscape, and one directed specifically toward the EndMT Cluster 5. This bifurcation indicates that EndMT commitment is a discrete fate decision made from within a proliferative endothelial state, rather than a gradual drift of the whole population. Pseudotime coloring (Fig. 6D) confirmed that Cluster 5 occupies high-pseudotime positions downstream of the cycling Cluster 4 origin. The convergence of two methodologically independent frameworks provides cross-validated evidence that the proliferative-to-EndMT transition is a directional, lineage-committed fate trajectory.

To quantify how TGF-β1 and TK22 shift the population across this landscape, we applied two-dimensional kernel density estimation (KDE) to the UMAP for each condition (Fig. 6E). In untreated controls, density was broadly distributed with no prominent EndMT concentration. TGF-β1 produced a strikingly focused, high-density domain in the lower UMAP region corresponding to Cluster 5, the topological signature of a stable EndMT cell-state attractor that emerges as the dominant state under profibrotic stimulation. TK22 co-treatment abolished this attractor, returning the distribution to a pattern closely resembling untreated controls. Because KDE provides a population-wide view orthogonal to individual feature plots, it explicitly demonstrates that TK22 prevents the emergence and stabilization of the EndMT cell state rather than causing a nonspecific loss of cell number or transcriptional diversity, a population-level precision consistent with its dual engagement of the anti-cell-cycle and anti-mesenchymal nodes.

Dot-plot analysis of curated EndMT-associated genes confirmed these findings transcriptomically across all three conditions (Fig. 6F). Under TGF-β1, Col1a1 (matrix), Il6 (inflammatory cytokine), Tagln/SM22 (cytoskeletal contractile), Trpv2 (mechanosensitive channel), and Vim (mesenchymal intermediate filament) were each upregulated by increased scaled expression and expanded expressing-cell fraction. TK22 substantially reduced all five toward control levels. The scope of this reversal, spanning matrix remodeling, inflammatory signaling, cytoskeletal reorganization, and mechanosensing, indicates that TK22 prevents the coordinated transcriptional program defining the mesenchymal state rather than suppressing isolated markers.

Beyond the primary Cluster 4-to-5 trajectory, scRNA-seq revealed a small but transcriptionally distinct Cluster 3, induced by TGF-β1 and attenuated by TK22 (Fig. 5A). Differential expression identified this cluster as a hypoxia and metabolic-stress endothelial signature (Egln3, Ndufa4l2, Cox4i2, Aldoc, Pfkfb3, Ddit4, Bnip3, Slc16a3, Pdk1). Its induction by TGF-β1 and suppression by TK22 links the pro-mesenchymal node directly to metabolic reprogramming: the AMPK-related kinases NUAK1 and SIK1/2/3 that TK22 inhibits are central regulators of metabolic and energy-stress signaling, providing a mechanistic bridge between the dual-node target set and the metabolic-stress program that accompanies profibrotic endothelial activation.

Collectively, UMAP clustering, PHATE-informed pseudotime, Monocle3 trajectory, KDE, and transcriptomic profiling resolve a coherent, directional mechanism: TGF-β1 drives a fate transition in which highly proliferative endothelial cells (Cluster 4) commit to an EndMT program (Cluster 5), accompanied by broad mesenchymal reprogramming and a secondary metabolic-stress state (Cluster 3). TK22 disrupts this conversion with marked cell-type specificity, selectively eliminating the EndMT-competent and stress-adaptive subpopulations while leaving the broader endothelial landscape intact. This precision distinguishes TK22’s dual-node action at the proliferative-to-mesenchymal fate branch point from global anti-proliferative or cytotoxic strategies and establishes single-cell trajectory analysis as a readout of cellular target engagement.

### TK22 prevents vascular remodeling in experimental PAH

**Figure 7.**
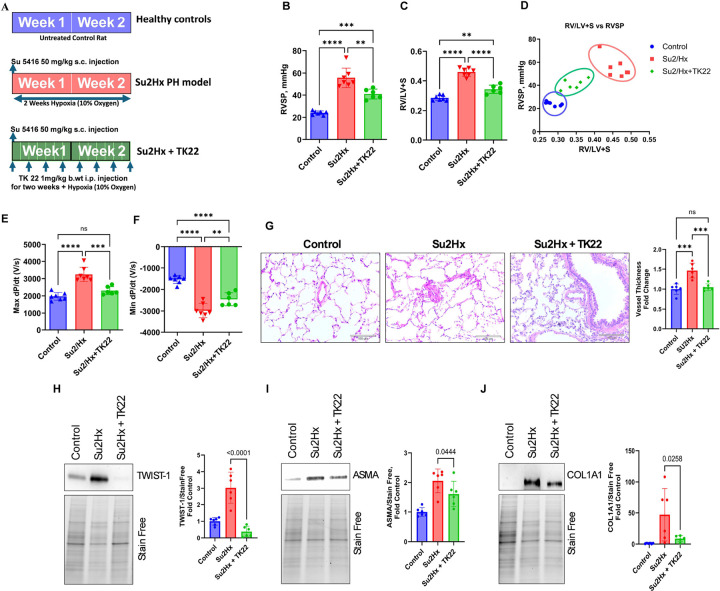
TK22 reverses EndMT and normalizes hemodynamics and vascular remodeling in the SuHx experimental PAH model. (A) Schematics showing in vivo animal experimentation. (B) Right ventricular systolic pressure (RVSP): Su2Hx elevated RVSP (~50–55 mmHg vs. 20–25 mmHg control, p < 0.05); TK22 significantly lowered RVSP. (C) Fulton index (RV/LV+S): significantly increased by Su2Hx and attenuated by TK22. (D) Scatter plot of RV/LV+S vs. RVSP showing a strong positive correlation across groups, with Su2Hx + TK22 shifting toward the control cluster. (E,F) Maximal (E) and minimal (F) rates of RV pressure change (MAX/MIN dP/dt), indexing systolic contractility and diastolic relaxation. MAX dP/dt was elevated in Su2Hx and normalized by TK22; MIN dP/dt was more negative in Su2Hx and returned toward control by TK22. (G) H&E morphometry of pulmonary arteries showing normalized vessel-wall thickness under TK22 relative to Su2Hx. (H-J) Lung-lysate immunoblots: Su2Hx upregulated TWIST-1 (G), which returned to control levels under TK22, and TK22 attenuated the elevated α-SMA (H) and COL1A1 (I). Data are mean ± SD; group comparisons by ANOVA with post hoc correction. *p < 0.05; **p < 0.01; ***p < 0.001; ****p < 0.0001.

In vivo efficacy testing was carried out targeting two weeks animal models grouped in three categories namely Control (untreated), Su2Hx, Su2Hx + TK22 (Fig. 7A). Animals treated with Su/Hx for two weeks (Su2Hx) showed elevated RVSP (Fig. 7B; ~50–55 mmHg vs. 20–25 mmHg in untreated control, p < 0.05), whereas TK22 co-administration significantly lowered RVSP relative to Su2Hx rats (p < 0.05). Su2Hx also significantly increased the RV/LV+S (Fulton index) ratio, which was significantly attenuated by TK22 (Fig. 7C). A scatter plot showed a strong positive correlation between RV/LV+S and RVSP across all groups (Fig. 7D), with the Su2Hx + TK22 group shifting toward the control cluster, indicating protection. Right ventricular function was likewise compromised in Su2Hx and restored by TK22: the maximal rate of RV pressure rise (MAX dP/dt), an index of systolic contractility, was significantly elevated in Su2Hx animals and normalized toward control by TK22, while the minimal rate (MIN dP/dt), an index of diastolic relaxation, was significantly more negative in Su2Hx and returned toward control values under TK22 (Fig. 7E–F; TK22 vs Su2Hx, p < 0.0001 for both indices, with TK22-treated MAX dP/dt statistically indistinguishable from control). Histological analysis of untreated control, Su2Hx, and Su2Hx + TK22 (1 mg/kg, every other day i.p. for two weeks) lungs revealed normalized vessel-wall thickness under TK22 relative to Su2Hx (Fig. 7F). Vascular remodeling by means of thickened vessel walls under Su2Hx were also brought back to control levels under TK22 administration (Fig. 7G). A protective effect against EndMT markers was seen in lung protein lysates: Su2Hx significantly upregulated TWIST-1, which returned to control levels under TK22 (Fig. 7H), and TK22 attenuated the elevated α-SMA and COL1A1 seen in Su2Hx lungs (Fig. 7I–J). Overall, TK22 consistently attenuated vascular remodeling via normalizing the EndMT lung signature, preventing disease progression in experimental PAH.

## Discussion

Lung endothelial cells maintain vascular homeostasis through hemostasis, gas exchange, barrier function, paracrine signaling, and regulation of vascular tone^26^. In pulmonary vascular disease, they can lose these functions and acquire maladaptive programs that drive vascular remodeling. A major manifestation of this plasticity is endothelial-to-mesenchymal transition (EndMT), in which endothelial cells downshift their identity while upregulating mesenchymal and cytoskeletal programs, gaining migratory and contractile properties that promote fibroproliferative remodeling 2. Because TGF-β signaling is a canonical driver of EndMT, TGF-β1 stimulation is widely used to induce and study this transition across endothelial lineages^27,28,29,30^. Our GFP-sorted mLECs showed a robust, time-dependent EndMT response to TGF-β1, providing a controllable and pure system to interrogate the regulators of endothelial fate conversion and, more importantly, to ask whether that conversion can be corrected pharmacologically at the level of the fate decision itself.

The central conceptual advance of this study is that EndMT is best understood, and best targeted, as a fate branch point rather than as a single signaling defect. Our single-cell analysis shows that TGF-β1 does not create a mesenchymal population de novo; it reroutes a committed proliferative endothelial subpopulation toward an EndMT fate. TGF-β1 expanded the EndMT-associated Cluster 5 while reducing the relative representation of highly cycling Mki67^+^ cells in Cluster 4, a reciprocal shift best explained not by anti-proliferative arrest but by accelerated state transition, as EndMT-associated transcripts (Tnnt2, Ptgs2, Serpine1) extended into the Cluster 4 region before cells gathered in the EndMT cluster. Two orthogonal trajectory frameworks converged on this interpretation: PHATE, which requires no predefined root, placed Cluster 4 at the earliest pseudotime with Cluster 5 immediately downstream, and Monocle3, initialized from the PHATE-informed root, formalized a bifurcating principal graph in which the proliferative origin diverges toward either the broader endothelial landscape or, under profibrotic stimulation, the EndMT state. Kernel density estimation reinforced this at the population level, revealing a focused, high-density EndMT cell-state attractor under TGF-β1 that was absent in controls. The proliferative-to-mesenchymal transition is therefore a directional, lineage-committed decision made within a cycling endothelial subpopulation; architecturally, it is a branch point at which the cell-cycle machinery that sustains the proliferative pool and the mesenchymal-signaling machinery that executes conversion intersect.

This architecture suggeststhat the most effective pharmacology would act at both arms of the branch point simultaneously, and TK22 does exactly that. TK22 originated as a structure-based, ATP-competitive CDK5 inhibitor, and by that design metric, it succeeded, inhibiting CDK5 with an IC_50_ of 181 nM. But unbiased profiling across 394 kinases showed that CDK5 is not its primary target; CDK5 complexes ranked 96th and 113th in the panel, and TK22 strong activity instead converges on two functionally coherent nodes. The first is an interphase cell-cycle node (CDK2, CDK3, CDK4, CDK6), which governs the cycling endothelial pool from which EndMT emerges. The second is a pro-mesenchymal node built around the AMPK-related kinases NUAK1 and SIK1/2/3, together with PDGFRβ and MLK3, which map onto TGF-β transcriptional output, YAP/SMAD fibrogenic reinforcement, non-SMAD MAPK/JNK signaling, and PDGF/TGF-β remodeling crosstalk. The coincidence of these two targets in a single molecule allows TK22 to disrupt the fate decision at their intersection: it constrains the proliferative substrate while simultaneously blocking the mesenchymal execution program. This is the mechanistic basis of branch-point targeting, and it is a conclusion that only unbiased profiling could deliver. A single-target reading of TK22 would have misattributed its action to CDK5 and missed the polypharmacology that explains it.

This phenotype is not a single-target CDK5 and is reinforced by the inhibitor comparisons. A dedicated commercial CDK5 inhibitor, PHA-793887, failed to reproduce TK22’s reversal of EndMT markers and unexpectedly elevated calponin, while the promiscuous inhibitor - Compound 1, only partially overlapped. If CDK5 inhibition were sufficient to reverse EndMT, a selective CDK5 inhibitor should have matched TK22. The efficacy therefore tracks TK22’s broader dual-node engagement rather than CDK5 blockade.

The cell-type specificity of TK22’s action is a defining feature with direct translational implications. Rather than broadly suppressing proliferation or inducing cytotoxicity, effects that would impair normal vascular homeostasis, TK22 selectively eliminated the EndMT-competent Cluster 5 and reduced EndMT-initiating cells within Cluster 4, while leaving the transcriptionally distinct endothelial clusters intact. This selectivity was evident across modalities: the TGF-β1-induced density attractor was abolished, and transcriptomic reversal spanned matrix remodeling (Col1a1), inflammatory signaling (Il6), cytoskeletal organization (Tagln, Vim), and mechanosensing (Trpv2), indicating suppression of the coordinated mesenchymal program rather than of isolated markers. Functionally, TK22 restored angiogenic tube formation and abolished acquired smooth-muscle-like contractility, and it returned TGF-β1-driven hyperproliferation to baseline without suppressing the normal growth of untreated endothelial cells. This point is the clearest signature of dual-node action: a general antiproliferative agent would suppress all endothelial growth, whereas coordinated cell-cycle-node inhibition acts on the pathologically activated, EndMT-routing pool. The population-level precision, disrupting a pathological fate conversion at a defined branch point while preserving endothelial heterogeneity, would be invisible to bulk transcriptomic or proteomic analysis and underscores the value of single-cell resolution in evaluating vascular-targeted therapeutics.

The two nodes also converge on the metabolic dimension of EndMT. TK22 attenuated a TGF-β1-induced hypoxia/metabolic-stress subpopulation (Cluster 3; Egln3, Pfkfb3, Pdk1, Bnip3, and related transcripts), a program that is mechanistically coupled to the pro-mesenchymal node: the AMPK-related kinases NUAK1 and SIK1/2/3 are central regulators of metabolic and energy-stress signaling. Their inhibition provides a direct link between TK22’s target set and the suppression of the metabolic-stress state that accompanies profibrotic endothelial activation, folding the metabolic reprogramming of EndMT into the same dual-node mechanism rather than treating it as a separate phenomenon. Consistent with cellular efficacy, TK22 normalized RVSP, Fulton index, RV systolic and diastolic function, vessel-wall thickness, and EndMT-associated protein expression (TWIST-1, α-SMA, COL1A1) in the Su5416/hypoxia rat model, translating branch-point disruption into hemodynamic and structural rescue in vivo.

### Limitations:

TK22 is a polypharmacology agent, and its inhibitory profile extends beyond the two named nodes: additional kinases, including AMPK holoenzyme complexes and hits such as FLT3, SYK, MAP4K2, and others, were also strongly inhibited at 10 μM. We frame the two nodes as the coherent driver set that most commonly explains the observed phenotype, not as the compound’s complete target list, and we cannot rule out contributions from off-node targets. Second, the dual-node mechanism is a strong inference from the convergence of kinome profiling and phenotype; it is not established by individual loss-of-function in each constituent kinase. Genetic or selective pharmacological dissection of NUAK1, the SIK family, PDGFRβ, MLK3, and the cell-cycle CDKs, individually and in combination, will be required to quantify the contribution of each node and to test whether co-inhibition is necessary and sufficient to disrupt fate-branch points. Third, profiling was performed at a single concentration (10 μM TK22/ 1 mM ATP). Importantly, TK22 can also bind other ATP-binding proteins beyond kinases, and future studies are needed to identify the full range of intracellular proteins that TK22 can target. These studies can be done in the future using the thermal proteome profiling technique, which is well-precedented for kinase inhibitors^31^. Fourth, pharmacological inhibition by TK22 may not fully recapitulate the phenotypes observed in genetic knockout models, as acute and reversible multi-kinase inhibition differs fundamentally from permanent gene deletion, which can induce developmental compensation and signaling network rewiring. Finally, CDK5 engagement is not excluded as a contributor, and the in vitro-to-in vivo translation of the precise cell-state effects remains to be resolved at single-cell resolution in diseased tissue.

## Conclusion

This study establishes the proliferative-to-mesenchymal fate branch point as a pharmacologically tractable control point in pulmonary vascular disease. Using GFP-sorted primary mLECs and single-cell resolution, we show that TGF-β1-driven EndMT is a directional conversion of a cycling endothelial subpopulation into a stable, committed mesenchymal cell-state attractor, resolved concordantly by UMAP clustering, PHATE embedding, Monocle3 pseudotime, and kernel density estimation. TK22, designed as a CDK5 inhibitor but revealed by unbiased profiling across 394 kinases to be a dual-node polypharmacology agent that co-inhibits the interphase cell-cycle kinases (CDK2/3/4/6) and a pro-mesenchymal AMPK-related kinase node (NUAK1, SIK1/2/3) together with PDGFRβ and MLK3, reverses this transition across morphological, proteomic, functional, and transcriptomic readouts, and normalizes hemodynamics and vascular remodeling in experimental pulmonary hypertension. Its defining property is precision: TK22 erases the EndMT-competent and metabolic-stress subpopulations while sparing normal endothelial growth and heterogeneity, a selectivity that follows from coordinated engagement of both arms of the fate branch point rather than from inhibition of any single kinase.

These findings reframe pharmacologic EndMT control. Rather than seeking selectivity for one regulator, we show that high cell-state selectivity can be achieved by coordinated suppression of the cell-cycle and mesenchymal kinase programs that intersect at a pathological fate decision, a dual-node polypharmacology principle that generalizes beyond TK22. Importantly, a similar effect can be achieved by co-treatment with kinase inhibitors that target cell cycle or mesenchymal kinase programs separately; however, having one compound with the required polypharmacological properties makes it easier to dose, formulate, and track drug metabolism than keeping track of two or more compounds that can, in addition, display drug-drug interactions with each other. This nominates TK22 as a mechanistically precise lead for EndMT-driven vascular disease and establishes single-cell trajectory analysis as an essential readout of cellular target engagement and cell-state precision in endothelial-targeted therapeutics.

## Material and Methods

### Experimental Animal Model:

Wild-type C57BL/6 mice and endothelial GFP reporter mice (Tg(TIE2GFP)287Sato/J; The Jackson Laboratory, Bar Harbor, ME, USA) were used for primary endothelial cell isolation. Female CD (Sprague-Dawley) rats (10–12 weeks old; n = 6–7 per group) were used for in vivo studies. Animals were housed under specific pathogen-free conditions with free access to standard chow and water and maintained on a 12-h light/dark cycle.

For mechanistic studies, pulmonary arterial hypertension (PAH) was induced in CD rats by a single subcutaneous injection of SU5416 (50 mg/kg), followed by exposure to normobaric hypoxia (10% O_2_) for 3 weeks and recovery under normoxic conditions for 2 weeks (Su5 model). Age-matched normoxic rats served as controls. Lung tissues were harvested at the end of the 5-week protocol and used for Western blot analysis of endothelial-to-mesenchymal transition (EndMT)-associated proteins.

For therapeutic studies, PAH was induced in CD rats by a single subcutaneous injection of SU5416 (50 mg/kg), followed by continuous exposure to normobaric hypoxia (10% O_2_) for 2 weeks (Su2 model). Age-matched normoxic rats served as controls. Rats in the treatment group received TK22 (1 mg/kg, intraperitoneally) every other day throughout the 2-week SU5416/hypoxia protocol.

At the experimental endpoint, rats were anesthetized with 2–3% isoflurane in oxygen delivered through a nose cone using a SomnoFlo^®^ low-flow electronic vaporizer (Kent Scientific Corporation, Torrington, CT, USA). Following surgical exposure of the right jugular vein, right ventricular systolic pressure (RVSP) was measured by advancing an SPR-513 pressure catheter (Millar Instruments, Houston, TX, USA) into the right ventricle. Hemodynamic data were acquired using a PowerLab 4/35 data acquisition system (AD Instruments, Colorado Springs, CO, USA). The maximal and minimal rates of right ventricular pressure change (Max dP/dt and Min dP/dt) were calculated from RV pressure waveforms using LabChart software (AD Instruments) as indices of right ventricular systolic contractility and diastolic relaxation, respectively.

Following hemodynamic assessment, the lungs were perfused with 0.9% NaCl through the RV before excision of the heart and lungs. RV hypertrophy was quantified using the Fulton index, calculated as the ratio of the RV free wall weight to the combined weight of the left ventricle and interventricular septum [RV/(LV+S)]. The left lung was fixed in 10% neutral-buffered formalin, embedded in paraffin, and sectioned for histological analysis, whereas the remaining lung tissue was snap-frozen in liquid nitrogen and stored at −80°C for protein expression studies.

Pulmonary vascular remodeling was assessed on deparaffinized 5-μm hematoxylin and eosin (H&E)-stained lung sections prepared by the IU School of Medicine Histology Core Facility (Indianapolis, IN). Morphometric analysis was performed on five to six randomly selected pulmonary arteries (50–300 μm in diameter) from each animal. Pulmonary arterial medial wall thickness was quantified using Fiji ImageJ software (version 1.52p; National Institutes of Health, Bethesda, MD). All morphometric analyses were performed by an investigator blinded to the experimental groups.

All animal procedures were approved by the Institutional Animal Care and Use Committee (IACUC) of Indiana University School of Medicine (Protocol No. 23077) and, where applicable, by the University of Arizona Institutional Animal Care and Use Committee (Protocol No. 15–579). All experiments were conducted in accordance with the National Institutes of Health Guide for the Care and Use of Laboratory Animals.

### Isolation and Culture of Primary Mouse Lung Endothelial Cells

Primary mouse lung endothelial cells (mLECs) were isolated from GFP reporter mice (Tg(TIE2GFP)287Sato/J) and wild-type C57BL/6 mice (12–13 weeks old; n = 5 per group) by fluorescence-activated cell sorting (FACS). Mice were euthanized in accordance with approved institutional animal care protocols, and lungs were aseptically excised, minced, and enzymatically digested in a solution containing collagenase type IV (1 mg/mL; Worthington Biochemical, LS004188), neutral protease (1 mg/mL; Worthington Biochemical, LS02109), and DNase I (1 mg/mL; Roche, 82672500) at 37°C for 45 minutes with gentle agitation.

Enzymatic digestion was terminated by adding Dulbecco’s Modified Eagle Medium (DMEM) supplemented with 10% fetal bovine serum (FBS). The resulting cell suspension was sequentially filtered through 70-μm (Corning, CLS431751) and 40-μm (Corning, CLS431750) cell strainers to obtain a single-cell suspension suitable for FACS. Lung cells isolated from wild-type mice were used as GFP-negative controls for gating, whereas GFP reporter mice provided GFP-positive endothelial cells for sorting (Fig. 3A).

GFP-positive endothelial cells were isolated using a BD FACSAria^™^ Fusion SORP cell sorter (BD Biosciences), seeded onto 0.1% gelatin-coated six-well plates, and cultured in endothelial cell medium (ScienCell, #1001) supplemented with 5% fetal bovine serum and 1% antibiotic-antimycotic solution (Thermo Fisher Scientific, #15240062). Cells were expanded and used at passage 4 for all subsequent experiments.

Endothelial identity was confirmed by characteristic cobblestone morphology and immunofluorescent staining for von Willebrand factor (vWF). The isolated mLECs were subsequently used for all downstream molecular and functional analyses (Fig. 3B–C).

### Design, synthesis, evaluation, and kinome profiling of TK22

#### Design:

TK22 was designed by structure-based drug design to achieve an ATP-competitive binding mode in CDK5, the target that anchored the medicinal-chemistry campaign. The crystal structure of CDK5/p25 (PDB ID: 7VDP)^32^ was used for docking studies with AutoDock 4. Protein structures were prepared by removing heteroatoms and water molecules, adding hydrogens, and assigning Kollman charges. The ligand was built in Avogadro and prepared in .pdbqt format with torsional flexibility. Docking was carried out using a grid box centered on the ATP-binding site, and the Lamarckian Genetic Algorithm was applied (100 runs, 2.5 million energy evaluations). The lowest-energy pose (−8.79 kcal/mol) (Fig. 2C) predicted hydrogen bonding with residues Cys83, Asn131, and Gln130, along with hydrophobic interactions involving Phe80, Val18, Leu133, and Ala143, supporting the binding stability of TK22. All molecular visualization and interaction analyses were performed using PyMOL (Fig. 2A) and Discovery Studio Visualizer (Fig. 2B). Because this design was anchored to a single target, the compound was subsequently subjected to unbiased broad-panel kinome profiling (below) to define its true target space rather than assume selectivity for CDK5.

#### Synthesis of TK22:

TK22 was synthesized in a three-step sequence (Fig. 2D) starting from 2-chloro-7H-pyrrolo[2,3-d] pyrimidine (1). In the first step, alkylation with tert-butyl 4-(bromomethyl) piperidine-1-carboxylate (2) afforded intermediate (3)^33^. The second step involved a Buchwald-Hartwig amination^18^ with aniline in the presence of a palladium catalyst to yield intermediate 5. Finally, deprotection with 4 M HCl in dioxane^19^ produced TK22 as the hydrochloride salt. The overall yield of the final compound was high (93%), and its structure and purity were confirmed by 1H and 13C NMR as well as high-resolution mass spectrometry, and purity of 97% by HPLC.

#### Biological Evaluation of TK22:

Inhibition of CDK5/p35 was assessed using the Kinase-Glo Plus Luminescent Kinase Assay (Promega, Cat. #V3771) 30 (Fig. 2E). Kinase reaction (56 μL) contained assay buffer (40 mM Tris-HCl, pH 7.5; 20 mM MgCl_2_; 0.1 mg/mL BSA; 0.1 M DTT), 2.5 μL enzyme (5 ng/μL), ATP (100 μM), Histone H1 substrate (1 μg/μL), and TK22 at concentrations ranging from 1 μM to 1.37 nM. After incubation for 1 h at 24 °C, 28 μL Kinase-Glo reagent was added to stop the reaction. Luminescence was measured on a Synergy H1 plate reader (BioTek), and data were analyzed with GraphPad Prism 10.0. TK22 inhibited CDK5 with an IC50 of 181.3 nM.

In addition to TK22, two reference inhibitors were used: compound 1^34^, a promiscuous covalent kinase inhibitor (500 nM; PF-6808472, Sigma-Aldrich), and PHA-793887, a commercial CDK5 inhibitor (5 nM; S1487, Selleckchem). These comparators were included to distinguish TK22's dual-node activity from single-target CDK5 inhibition.

#### Kinome profiling:

To define the full target space of TK22 without assuming selectivity for its design target, the compound was profiled against a panel of 394 recombinant protein kinases (AssayQuant Technologies) using a Sox-based continuous kinetic (PhosphoSens) fluorescence assay. TK22 was tested at a single concentration of 10 μM in the presence of 1 mM ATP, with 1% DMSO as the vehicle control; reactions (10 μL final volume) were run at 30 °C, and the initial reaction velocity for each kinase was compared to its DMSO control to calculate percentage inhibition. Rate-replicate variability was expressed as the standard error of the mean divided by the mean (×100); enzymes for which one replicate was rejected are reported without a variability value. Kinases were ranked by percentage inhibition, and hits were grouped by Manning kinome family (Fig. 2F–G). A 70% inhibition threshold was used to define strong hits, with a secondary 90% threshold to define the most potent targets.

### Endothelial-to-Mesenchymal Transition (EndMT) Induction

Mouse lung endothelial cells (mLECs) at passage 4 were subjected to EndMT induction through treatment with recombinant transforming growth factor-beta 1 (TGF-β1; PeproTech Inc., Cranbury, NJ, USA) at a concentration of 1 ng/ml. Cells were incubated under these conditions for specified durations (4 hours, day 3 and day 5), with control groups receiving complete extracellular matrix (ECM) medium without TGF-β1 (Fig. 3D–F). Media was replenished every other day, and morphological transformations characteristic of EndMT, such as spindle-shaped phenotype and loss of cobblestone morphology, were monitored via phase-contrast microscopy.

To test whether TK22 attenuates EndMT, cells were co-treated with TK22 at 2 μM and 5 μM in combination with TGF-β1. For comparative evaluation, cells were also treated with two reference kinase inhibitors: compound 1 (a promiscuous kinase inhibitor) and PHA-793887 (a commercial CDK5 inhibitor) (Fig. 3G–I), which serve to dissociate EndMT reversal from single-target CDK5 inhibition. Following preliminary dose-response assessment, the optimal TK22 concentration was determined and used for subsequent experiments. Both treated and untreated groups were analyzed for EndMT-specific markers, including α-smooth muscle actin (αSMA) (14-9760-82, Invitrogen, Waltham, MA, USA), SM22α (40471, Cell Signaling Technology, Danvers, MA, USA), and Calponin (CALP) (17819, Cell Signaling Technology) via immunocytochemistry and/or quantitative gene expression analyses.

This approach facilitated the assessment of TK22’s capacity to attenuate TGF-β1-induced EndMT, thereby elucidating its potential as a therapeutic agent in vascular remodeling and related pathologies.

### Western Blotting Analysis of EndMT Marker Expression in GFP-mLECs

GFP-expressing mouse lung endothelial cells (GFP-mLECs) subjected to various induction conditions were harvested at specified time points (4 hours, day 3, and day 5). Cells were lysed using RIPA buffer (Thermo Scientific, Rockford, IL, USA) supplemented with a protease inhibitor cocktail (Thermo Scientific, 78429). Protein concentrations were accurately quantified using the Microplate BCA Protein Assay Kit (Pierce Biotechnology, Rockford, IL, USA).

Equal amounts of total protein (10μg per sample) were mixed with 6x Laemmli sample buffer (Boston Bioproducts Inc., Ashland, MA, USA) and denatured by heating at 95°C for 5 minutes. Protein samples were then resolved by SDS-PAGE using 4–20% Mini-PROTEAN TGX Stain-Free^™^ gels (Bio-Rad Laboratories, Hercules, CA, USA). Proteins were transferred onto PVDF membranes via the Trans-Blot Turbo Transfer System (Bio-Rad). Membranes were blocked with Everyblot Blocking Buffer (Bio-Rad, #12010020) to prevent nonspecific binding.

Membranes were probed overnight at 4°C with primary antibodies targeting EndMT-associated markers, SM22α (Cell Signaling, #40471, 1:1000), α-SMA (Invitrogen, #14-9760-82, 1:1000), Calponin-1 (Cell Signaling, #17819, 1:1000), FOXC2 (Proteintech, #23066–1-AP, 1:1000), TRPV2 (Proteintech, #15991–1-AP, 1:1000), COL1A1 (Cell Signalling, #72026, 1:1000), and SERPINE1 (Abcam, #ab222754, 1:1000). Following primary incubation, membranes were incubated with HRP-conjugated secondary antibodies—Anti-Rabbit IgG (Cell Signaling, #7074S, 1:5000) and Anti-Mouse IgG (Cell Signaling, #7076S, 1:5000) for 1 hour at room temperature.

Chemiluminescent detection was performed using the ChemiDoc^™^ MP Imaging System (Bio-Rad), and band intensities were quantified with Image Lab^™^ software. Stain-Free gel images were utilized to normalize protein loading across samples, following established protocols^35^.

### Immunocytochemistry for EndMT Marker Detection

Endothelial cells were seeded onto sterile coverslips (NC0326897, Fisher Scientific) and subjected to induction with 1 ng/ml TGF-β1, either alone or in combination with TK22 (5 μM), for 5 days. Following treatment, cells were fixed with 4% paraformaldehyde in PBS for 15 minutes at room temperature. Permeabilization was performed with 0.2% Triton X-100 (Thermo Fisher Scientific), and nonspecific binding sites were blocked with 15% bovine serum albumin (BSA) in PBS for 1 hour at room temperature.

After blocking, cells were incubated overnight at 4°C with primary antibodies targeting EndMT markers: SM22α (Cell Signaling, #40471, 1:100), α-SMA (Invitrogen, #14-9760-82, 1:100), and Calponin-1 (Cell Signaling, #17819, 1:100). The following day, after five washes with PBS, cells were incubated with appropriate secondary antibodies—Donkey anti-Rabbit IgG Alexa Fluor 647 (Invitrogen, #A32795, 1:300) and Donkey anti-Mouse IgG Alexa Fluor 647 (Invitrogen, #A32787, 1:300), for 1 hour at room temperature in the dark.

Finally, coverslips were mounted inversely onto microscope slides using ProLong^™^ Diamond Antifade Mountant with DAPI (ThermoFisher Scientific, #P36962) to counterstain nuclei. Imaging was conducted with a Revolve Fluorescence Microscope (Echo, San Diego, CA). Based on preliminary analysis, the inhibitor showing the most effective suppression of EndMT marker expression (TK22) was selected for subsequent comprehensive studies.

### Cell Proliferation Assay

Cell proliferation across all experimental groups was assessed at three distinct time points, day 1, day 3, and day 5, using the ApexBio Cell Proliferation Kit (ApexBio, K1018). For each condition, 10,000 cells were seeded per well in a 96-well culture plate. Following cell attachment and growth, 10 μl of the CCK-8 reagent was added to each well containing 100 μl of complete ECM media, in accordance with the manufacturer's instructions.

The plates were then incubated at 37°C in a humidified incubator with 5% CO_2_ for 4 hours. Absorbance was subsequently measured at 450 nm using a microplate reader (Model: Infinite 200 Pro, Tecan, Austria). The optical density values served as an indicator of cell viability and proliferation rate at each designated time point.

### Angiogenesis (Tube Formation Assay)

The ability of endothelial cells to undergo angiogenesis, a hallmark feature of endothelial functionality, was assessed using a matrigel-based tube formation assay following a previously established protocol^36^. Briefly, Matrigel^®^ (Millipore Sigma, E6909, Darmstadt, Germany) was thawed on ice and evenly coated onto 96-well plates at 50μl per well, then allowed to solidify in a humidified incubator with 5% CO_2_ at 37°C for 20–30 minutes.

Subsequently, 35,000 cells from each group—namely, untreated control, and cells cultured for 5 days with 1 ng/ml TGF-β1 with or without 5μM TK22—were seeded onto the precoated wells. Cells were monitored over the course of 5 hours, and images of tube-like structures were captured using an ECHO microscope.

To ensure reproducibility and enable statistical comparison, the experiment was performed with six biological replicates (n=6). The extent of tube formation was analyzed qualitatively and quantitatively to evaluate functional angiogenic capacity across different treatment groups.

### Collagen Gel Contraction Assay

The contractile capability of cells from all experimental groups (control, 1 ng/ml TGF-β1, and 1 ng/ml TGF-β1 combined with 5 μM TK22) was evaluated using a collagen gel lattice contraction assay, following established protocols^23,37^.

Briefly, 1 × 10^5^ cells from each group were mixed with rat tail collagen type I solution (final concentration 1 mg/ml; Gibco, Life Technologies), supplemented with NaOH (1 M; Sigma), 0.1% acetic acid, and ECM components to generate a homogeneous collagen cell suspension. Aliquots of 500 μl from each suspension were carefully pipetted onto 24-well tissue culture plates (229124, CELLTREAT, Ayer, Massachusetts, USA) and allowed to polymerize at room temperature for 20–30 minutes.

Once gelation was complete, the gels were gently detached using a 200μl pipette tip, followed by the addition of 500μl serum-free ECM media, with or without 80 mM KCl. Plates were then incubated at 37°C in a humidified 5% CO_2_ atmosphere.

Gel contraction was monitored at specific time intervals (0, 3, and 24 hours), with images captured using a KEYENCE microscope (BZ-X810, Itasca, IL, USA). Quantitative analysis of gel contraction was performed by measuring the gel area in each image using ImageJ software (Version 1.54m). The extent of contraction was expressed as a percentage reduction in gel area relative to the initial (0 hour) measurement.

Once gelation was complete, the gels were gently detached using a 200μl pipette tip, followed by the addition of 500μl serum-free ECM media, with or without 80 mM KCl. Plates were then incubated at 37°C in a humidified 5% CO_2_ atmosphere.

Gel contraction was monitored at specific time intervals (0, 3, and 24 hours), with images captured using a KEYENCE microscope (BZ-X810, Itasca, IL, USA). Quantitative analysis of gel contraction was performed by measuring the gel area in each image using ImageJ software (Version 1.54m). The extent of contraction was expressed as a percentage reduction in gel area relative to the initial (0 hour) measurement.

### Single Cell RNA Sequencing (scRNA-seq): Sample preparation, data processing and analysis

For scRNA-seq, GFP-mLECs at passage 3 were segregated into three experimental groups namely untreated control (UT), 1ng/ml TGF-β1 treated (TGF-β1) and 1ng/ml TGF-β1 + 5μM TK22 treated (TGF-β1 + TK22) under three days induction protocol. Afterwards, sample preparation was done with GFM-X Flex Sample preparation v2 kit following manufacturer’s instructions (1000781, 10x Genomics, Pleasanton, CA, USA). Briefly, after induction for three days, cells were trypsinized, pelleted (centrifugation at 400 rcf for 5 minutes at 4°C), and incubated in fixation buffer at 4°C for 16–24 hrs followed by centrifugation at 850 rcf for 5 minutes at room temperature. After supernatant removal, quenching buffer containing 50% glycerol was added and all samples were stored at − 80°C until further use.

Gene expression count matrices were generated using the Cell Ranger Multi pipeline (10x Genomics) implemented on a high-performance computing (HPC) cluster, following the manufacturer’s recommendations for Flex sample preparation and barcode demultiplexing. Downstream analyses were performed in R (v4.3.1) using the Seurat package (v5.2.0).

Quality control filtering was applied to remove low-quality cells based on gene and transcript abundance and mitochondrial RNA content. Cells with fewer than 800 detected UMIs or greater than 50,000 UMIs were excluded from further analysis. Additionally, cells expressing fewer than 200 or more than 7,500 genes were removed. Cells with mitochondrial transcript content exceeding 30% of total UMIs were excluded to minimize the contribution of stressed or dying cells.

After quality control, gene expression data were normalized using a standard log-normalization approach, and 2,000 highly variable genes were identified using the FindVariableFeatures function. Datasets were integrated using an anchor-based integration strategy implemented in Seurat to correct for batch effects across experimental conditions. Principal component analysis (PCA) was performed on the integrated dataset using 50 principal components derived from the variable genes, and significant components were selected for downstream analysis.

Unsupervised clustering was performed by constructing a shared nearest neighbor (SNN) graph followed by graph-based clustering using a resolution of 0.5. Uniform Manifold Approximation and Projection (UMAP) was applied for dimensionality reduction using the first 30 principal components and for visualization of cell clusters.

Trajectory analysis is a computational method to reconstruct dynamic biological processes from single-cell data by ordering cells along a continuous path, known as pseudotime, reflecting a cell’s relative position along a biological progression.

To ensure robust characterization of cellular dynamics, two trajectory approaches were employed: PHATE (Fig. 6A–B) and Monocle3 (Fig. 6C–D). Monocle 3 is one of the most widely used trajectory inference tools^38,39^; however, in Monocle3, to establish the order of cells and calculate pseudotime, it is necessary to select a starting node. On the other hand, PHATE provides a structure that enables the identification of the trajectory root^40^. Therefore, we used PHATE to capture the global manifold structure, visualize continuous transitions between cell states, and determine the root. Based on the PHATE embedding, the trajectory root (starting point) was defined to represent the inferred origin of the biological process. This PHATE-informed root was subsequently used to initialize pseudotime ordering in Monocle3 where cells were ordered along a principal graph representing transcriptional progression. Trajectories were inferred independently by each method, enabling comparison of cellular transitions while maintaining a consistent starting point across analyses. The use of both methods allows cross-validation of inferred transitions, combining structure-preserving visualization with quantitative trajectory modeling.

Kernel Density Estimation is a method to estimate the probability density distribution of data by smoothing individual data points. Density distributions of cells in UMAP space were estimated for each condition using two-dimensional kernel density estimation (KDE).

The resulting density contours were used to identify regions of highest cellular concentration, representing the most stable and dominant cell states within each condition (Fig. 6E).

Feature plots and cluster-based visualizations (Fig. 6F) were generated to assess transcriptional differences between untreated, TGF-β1-treated, and TGF-β1 + TK22-treated endothelial cells.

### Statistical Analysis

All experimental data were analyzed using GraphPad Prism version 10 (GraphPad Software, San Diego, CA, USA). Prior to analysis, outliers were identified using Grubbs’ test via the GraphPad outlier calculator (α = 0.5; https://www.graphpad.com/quickcalcs/Grubbs1.cfm). Data are presented as mean ± standard deviation (SD). For comparisons between two groups, unpaired t-tests were performed to determine statistical significance. Multiple group comparisons were conducted using one-way ANOVA and two-way ANOVA followed by Tukey’s and Sidak’s or Bonferroni post hoc tests to assess differences among groups. A p-value less than 0.05 was considered statistically significant, with all tests applied at a 95% confidence interval.

## Supplementary Material

Supplementary Files

This is a list of supplementary files associated with this preprint. Click to download.

- GraphicalAbstract.tif
- Supplementarydata1.docx
- Supplementaldata2.docx

**Graphical Abstract.** A four-panel schematic summarizing the study: (1) EndMT as a proliferative-to-mesenchymal fate branch point; (2) single-cell trajectory revealing a stable EndMT cell-state attractor; (3) TK22 acting at the branch point through dual kinase-node inhibition (Node 1, anti-cell-cycle CDK2/3/4/6; Node 2, anti-mesenchymal NUAK1/SIK1/2/3, PDGFRβ, MLK3); and (4) reversal of pulmonary hypertension in vivo.

## Abbreviations

α-SMA: Alpha Smooth Muscle Actin
ANOVA: Analysis of Variance
BCA: Bicinchoninic Acid Assay
CALP: Calponin
CDK: Cyclin-Dependent Kinase
DMEM: Dulbecco’s Modified Eagle Medium
DNase I: Deoxyribonuclease I
EC: Endothelial Cell
EndMT: Endothelial-to-Mesenchymal Transition
FACS: Fluorescence-Activated Cell Sorting
FBS: Fetal Bovine Serum
GFP: Green Fluorescent Protein
H&E: Hematoxylin and Eosin
IACUC: Institutional Animal Care and Use Committee
Max dP/dt: Maximum Rate of Right Ventricular Pressure Development
mLEC: Murine Lung Endothelial Cell
Min dP/dt: Minimum Rate of Right Ventricular Pressure Development
NIH: National Institutes of Health
NUAK1: NUAK Family Kinase 1
PAH: Pulmonary Arterial Hypertension
PDGFRβ: Platelet-Derived Growth Factor Receptor Beta
RV: Right Ventricle
RVSP: Right Ventricular Systolic Pressure
scRNA-seq: Single-Cell RNA Sequencing
SIK: Salt-Inducible Kinase
SU5416: Semaxanib (SU5416)
SuHx: Sugen/Hypoxia
TGF-β1: Transforming Growth Factor Beta 1
TK22: Cyclin-Dependent Kinase 5 Inhibitor 22
vWF: von Willebrand Factor
WT: Wild Type
